# Measuring the mismatch between field of study and occupation using a task-based approach

**DOI:** 10.1186/s12651-018-0243-y

**Published:** 2018-09-06

**Authors:** Mauricio Reis

**Affiliations:** Instituto de Pesquisa Economica Aplicada, Av. Presidente Antonio Carlos, 51(1409), Rio de Janeiro, RJ 20020-010 Brazil

**Keywords:** Occupations, Fields of study, Skill requirements, J24, J31, I26

## Abstract

This paper seeks to provide a continuous measure to represent the distance between skills acquired in tertiary education and those required in an individual’s occupation. This distance measure, which is computed by combining data from the 2010 Brazilian census with information from the 2010 Brazilian classification of occupations, suggests that workers usually classified in most of the literature into a single group of mismatches are in fact quite heterogeneous in the way their occupations are associated with areas of study. Evidence also shows that, even among mismatched workers, hourly labor earnings tend to decrease as the distance measure increases. This indicates the labor earnings penalty is not the same for all mismatched workers, seemingly changing substantially depending on the level of similarity between occupation and field of study.

## Introduction

Empirical evidence shows that individuals with tertiary education employed in occupations unrelated to their fields of study earn, on average, less than those in occupations closely matched to their fields of study. Part of this labor earnings penalty has been attributed to the fact that a share of the skills acquired during tertiary education could be specific to occupations related to the chosen field of study, and those who are mismatched may be inefficiently using their skills (Robst 2007).^1^

As also emphasized by Robst (2007), the extent of the earnings losses associated with the occupation-field of study mismatch depends on the degree of transferability of skills. Knowledge acquired in a given field of study should be more or less valuable depending on how these skills are useful in an individual’s occupation. Estimates are usually consistent with this hypothesis, as partially mismatched individuals, that is, those who hold a job that is somewhat related to their fields, usually earn more than completely mismatched ones but less than those workers who are strongly matched to their jobs (Robst 2007; Nordin et al. 2010).

The empirical approach usually adopted to estimate the labor earnings consequences of mismatches between occupation and field of study among graduates is based on binary variables to represent their statuses as matched, weakly matched, or mismatched. However, this approach does not seem to be able to appropriately represent disparities in the degree of similarity between occupations and fields of study. Even among workers classified as mismatched, there would be great heterogeneity in the way their occupations are related to their fields of study. A mismatched worker may have an occupation in which a substantial share of the skills acquired during tertiary education is still useful, whereas another mismatched worker who completed the same program may have an occupation that requires completely different skills from those associated with the type of tertiary program completed.

The aim of this paper is to provide a continuous measure to represent the distance between skills acquired in tertiary education and those required in an individual’s occupation. As shown by Gathmann and Schonberg (2010), human capital is at least partially transferable across occupations with similar task requirements, and workers who move to a distant occupation suffer a larger wage loss than those who move to occupations with similar skill requirements to those of their former occupations. However, task specialization may already begin prior to labor market entry (Sloane 2003; Rocher 2011). The approach adopted in this paper is similar to the one proposed by Gathmann and Schönberg (2010) to measure transferability of skills across occupations using a task-based approach, in which workers apply their skills to tasks required in their occupations to produce output.^2^ Nevertheless, the empirical analysis here investigates the portability of human capital from tertiary education to occupation. The distance measure presented in this paper intends to offer more information on the transferability of skills from tertiary education to labor market than the one provided by dummies for mismatch and weak match, helping to explain whether changes in labor earnings are related to differences in the level of the education-occupation mismatch.

The empirical analysis in this paper uses data from the 2010 Brazilian census and the 2010 Brazilian Classification of Occupations (Classificação Brasileira de Ocupações 2010). Making use of information from the latter data source, each 4-digit level occupation is represented by a mix of tasks usually performed by workers. Then, the distance measure between field of study and occupation is computed based on the similarity between tasks representing an individual’s occupation and tasks representing the occupation most closely related to his or her field of study. The distance measure can be imputed to each employed individual in the 2010 census dataset.

Census data reveal that about half of the Brazilian workers with tertiary education are classified as mismatched according to the classification based on binary variables. Evidence also indicates, however, there is a lot of heterogeneity in the way the occupations of those mismatched workers are associated with their corresponding areas of study. This can be illustrated by the fact that the proportion of mismatched workers in the lower tail of the distance measure distribution is almost the same as that of mismatched workers at the top of the distribution.

The results show that hourly labor earnings penalties tend to increase as the distance measure between occupation and field and study enlarges. It seems that, even among mismatched individuals, those who have occupations with similar skill requirements in their fields of study are more able to transfer skills acquired during tertiary education than workers who have occupations unrelated to their fields of study. Thus, estimates suggest that the continuous measure of mismatch presented here helps to capture an important part of the heterogeneity in the labor earnings differences between mismatched and matched workers that is not accounted for by binary variables.

The paper is structured as follows. Section 2 presents the data sources and describes the construction of the continuous distance measure between area of study and occupation. Section 3 presents the descriptive analysis of the data. Section 4 reports the empirical findings relating the distance measure to hourly labor earnings, whereas Sect. 5 has the main conclusions of the paper.

## Data

The analysis in this paper combines individual data from the 2010 census, conducted by the IBGE (*Instituto Brasileiro de Geografia e Estatística*), with information on occupations provided by the 2010 Brazilian Classification of Occupations (Classificação Brasileira de Ocupações 2010)—henceforth CBO—from the Brazilian Ministry of Labor. This section describes these two data sources and how they are combined to provide a continuous distance measure between individuals’ occupations and their fields of study.

### Occupations and tasks performed in the 2010 CBO

The 2010 CBO has detailed descriptions of 607 occupations at the 4-digit level regarding workers’ education and experience necessary to perform each occupation, work environment, tools and technology workers use in the occupation, personal characteristics that can affect workers’ performance, and activities that are usually performed by workers in the occupation. Those descriptions are based on the developing a curriculum (DACUM) method. The DACUM process builds on the assumptions that expert workers are the best ones to describe their jobs and that a job can be described through tasks performed by successful workers.

In the first step of the DACUM process, each occupation was analyzed by a panel of 8 to 12 expert workers for two working days. This team provides the occupation profile, which includes activities that workers must perform. The panel members of each occupation are selected by the Brazilian Ministry of Labor and are considered outstanding workers in their occupations. In the second step, each occupational profile is validated by another panel of expert workers during one working day. The overall process of occupation description comprises around 7000 workers.

Thus, information on job tasks are provided by job analysts instead of being based on self-reports of job holders, as in Gathmann and Schönberg (2010) and Spitz-Oener (2006). As argued by Handel (2016), job incumbents may overestimate their self-reports, whereas analysts usually have less close knowledge of jobs than do the employees themselves. This latter problem could be mitigated in the case of CBO, as panel members are themselves workers in the occupation, although they probably hold higher positions, as outstanding workers, which could also affect their evaluations.

Between 4 and 14 main activities are assigned to each occupation (on average, about eight different tasks are assigned to each occupation). Based on that information, activities are arranged in about 80 aggregated groups, which are combined into 18 task categories.^3^ Appendix Table 5 shows these 18 task groups.

The relative importance of task category *j* in occupation *k* is defined as:

1 $$Task_{jk} = \frac{number\;of\;activities\;assigned \;to\;category\;j\;in\;occupation\;k }{total\;number\;of\;activities\;in\;occupation\;k}.$$

According to this procedure, each occupation *k* can be characterized by an 18-dimensional task vector, where $\sum\nolimits_{j = 1}^{18} {Task_{jk} } = 1$ for k = 1, 2,…K. For an electronic engineer, for example, planning and designing activities represent 5 out of 7 tasks, whereas selling represents almost 50% of the tasks performed by sales workers, although tasks assigned to this latter occupation also include organizing, calculating, and packing and transporting.^4^

### The 2010 census

The 2010 census covers more than 10% of the Brazilian population and contains information on education and labor market, among many other variables. The survey provides information on workers’ occupations at the 4-digit level. Occupations in the 2010 census can be associated with their counterparts in the 2010 CBO. Thus, the measure of task content of each occupation in the 2010 CBO, as described in Sect. 2.1, can be imputed to employed individuals in the 2010 census. For individuals who completed tertiary education, the 2010 census also gives information on their fields of study.

Following the approach adopted by Nordin et al. (2010), based on direct comparisons between occupations and fields of study, workers can be classified into three groups. Individuals in occupations closely related to their fields of study are classified as matched, while those for whom the occupation is considered only partially related to the area of study are classified as weakly matched, and mismatched individuals are those who do not belong to the latter two groups.^5^

The 2010 census sample used in this paper comprises only employed individuals with a bachelor’s degree. Military and public workers are excluded from the sample, which is also limited to those aged between 25 and 60 years with information on hourly labor earnings, field of study, and occupation. Legislators, senior government officials, traditional chiefs and heads of villages, and senior officials of special-interest organizations are also dropped. Only those with positive labor earnings are included in the analysis. Information on field of study is defined according to the individual’s highest educational degree. Thus, individuals who have a master’s or a doctoral degree are also excluded because it is not possible to know whether they have a bachelor’s degree related or unrelated to their occupations. After all exclusions, the sample consisted of 554,638 individuals distributed into 422 occupations, defined at the 4-digit level, and into 79 fields of study.

### Measuring the distance between occupation and field of study

The next step in the empirical approach consists in attributing measures for task contents to each field of study. This way, each of the 79 fields of study is associated with its most closely related occupation, which is quite straightforward in most of the cases, and the task content of this occupation is assigned to the corresponding area of study. Each field of study is represented by only one 4-digit occupation. Although the assignment of the occupation most closely related to a given field of study has some degree of arbitrariness, classifying workers into matched, weakly matched, and mismatched is much more challenging, since the boundaries for these categories are usually unclear. For example, the occupation most closely associated with a degree in mechanical engineering is mechanical engineer, but it is not clear how an individual with a degree in mechanical engineering who works as a civil engineer or as a mechanical engineering technician would be classified. Appendix Table 6 shows the field of study-occupation matches, and the occupations defined as the most closely associated with each area of study.

According to the approach adopted here, an individual who completed a given degree accumulated human capital to perform tasks required to work in the occupation most closely related to his or her area of study. However, for part of those workers, in particular the mismatched ones, only a fraction of the acquired human capital would be useful in another occupation. The size of this fraction depends on the similarity between tasks associated with the worker’s field of study and those required in his or her occupation.^6^

The distance measure between field of study *m* and occupation *n* is computed by the uncentered correlation of their vectors of tasks (Jaffe 1986):^7^

2 $$Dist_{mn} = 1 - Angsep_{mn} ,\quad {\text{where}}\;Angsep_{mn} = \frac{{\mathop \sum \nolimits_{j = 1}^{18} task_{mj} \times task_{nj} }}{{\left[ {\left( {\mathop \sum \nolimits_{j = 1}^{18} task_{mj}^{2} } \right) \times \left( {\mathop \sum \nolimits_{j = 1}^{18} task_{nj}^{2} } \right)} \right]^{1/2} }}.$$

The distance measure ranges from 0 to 1. When the vector of tasks in occupation *n* is identical to the one in the occupation representing field of study *m*, *Dist*_*mn*_ is equal to 0. If the vectors of job contents representing an occupation and a field of study are completely different, the distance measure between them is equal to 1.

The distance measure in Eq. (2) is based on a number of other assumptions in addition to those already mentioned. The 2010 CBO provides a list of activities usually performed by workers in a given occupation, but there is no information on the intensity of use of each of them. Thus, it is assumed that all activities in a given occupation have the same weight. It is also assumed that all workers perform the tasks assigned to their occupations, although there would be a lot of variation across tasks performed by workers in the same 4-digit occupation. In addition, the distance measure does not take into account that some tasks are more similar than others, and treats all tasks symmetrically. Another assumption adopted to construct the distance measure between area of study and occupation is that a given task is similar across different occupations. This is a huge simplification, since tasks are occupation-specific in some cases. The use of binary variables for mismatched and weakly matched individuals allows us to consider these specificities, in spite of the subjective approach, which could be pointed out as an advantage relative to the continuous measure proposed here.^8^

As a robustness check, task contents by fields of study are also computed taking into account the whole set of occupations for which a course is classified as matched instead of only the one considered most closely related. In this case, the weight of a given task associated with a field of study is the ratio between the sum of activities classified in this task across all matched occupations and the sum of all activities in all matched occupations. The distance measure computed in this way is represented by Dist*.

## Descriptive statistics

Table 1 presents the descriptive statistics of the sample separately for individuals classified as matched, weakly matched, and mismatched, making use of dichotomous variables defined through comparisons between fields of study and occupations, similar to the approach adopted by Nordin et al. (2010). One-third of the workers with tertiary education in Brazil are in occupations classified as matched to their fields of study, while 11% are in occupations that can be considered only somewhat related to the individuals’ area of study, and more than half are mismatched. Among those in the latter group, 60% have an occupation that does not require tertiary education.

**Table 1:** Summary statistics of individuals in the sample. Source: the 2010 Brazilian census

	Matched	Weakly matched	Mismatched
	(1)	(2)	(3)
Monthly labor earnings (R$)	4066.7	2731.4	2847.0
Hourly labor earnings (R$)	120.2	83.6	86.6
Age	38.45	39.1	38.3
Female (%)	50.30	70.07	53.51
Black (%)	19.27	31.00	24.50
Unskilled occupation (%)			60.42
Distance measure	0.034	0.122	0.518
Observations	171,647	70,002	312,989
Weighted share (%)	33.33	10.90	55.77

The sample comprises employed individuals with a bachelor’s degree, aged between 25 and 60 years

Table 1 also reports that matched individuals earn about 40% more per hour than weakly matched and mismatched workers. The average hourly labor earnings are quite similar between the latter two groups. As also shown in Table 1, the percentages of women and black individuals are much higher among weakly matched workers,^9^ whereas mean age is similar across the three groups of workers.

The distance measure is very close to 0 for matched individuals, reaching an average value of 0.03. In fact, it is 0 for three quarters of the workers in this group. In most of the cases, this is a consequence of the approach used to associate fields of study with occupations.^10^ Among weakly matched workers, the distance measure has an average equal to 0.12 and a median equal to 0.06. Among workers classified as mismatched, both the average and the median of the distance measure are about 0.50.

**Fig. 1:**
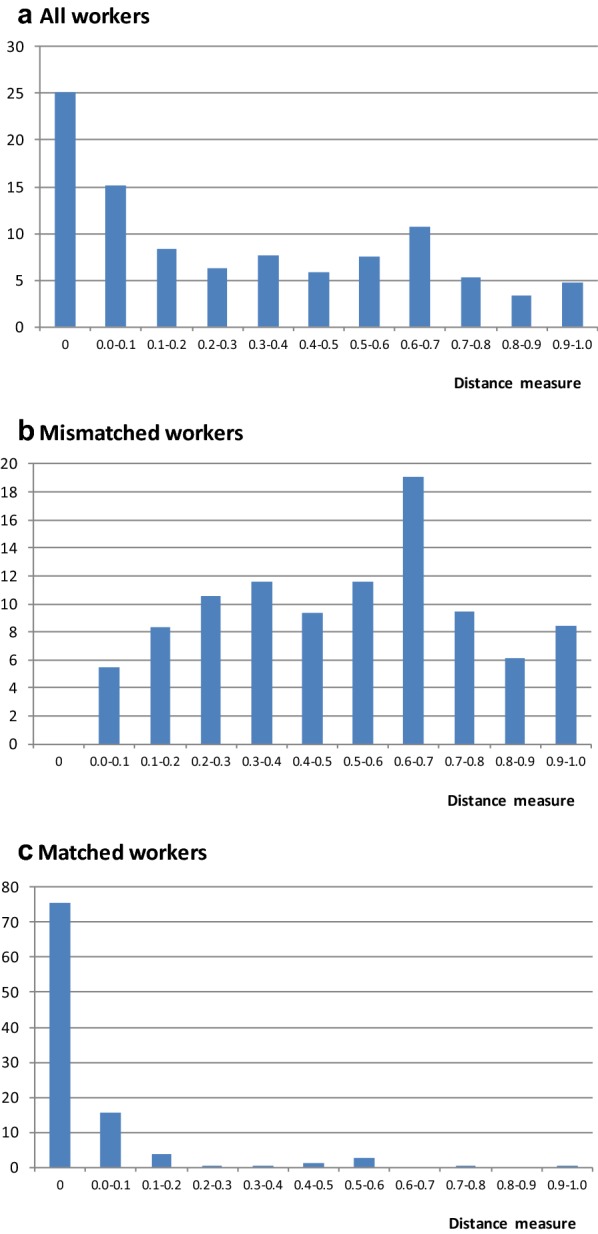
Distribution of workers across the distance measure between field of study and occupation. The sample comprises employed individuals with a bachelor’s degree, aged between 25 and 60 years. Source: the 2010 Brazilian census

Panel A of Fig. 1 presents the distribution of individuals in the sample for different intervals of the distance measure between area of study and occupation. The distance measure is equal to 0 for 25% of the individuals in the sample, almost all of them classified as closely matched, including those in occupations used as reference for their fields of study. The distance measure ranges between 0 and 0.1 for 15% of the workers, most of them classified as matched or weakly matched. The distribution of workers for whom the distance measure ranges between 0.1 and 1.0 is balanced across the whole intervals.

Panel A of Fig. 1 reveals great heterogeneity among mismatched workers, who represent most of those for whom the distance measure is higher than 0.2. This is reinforced by Panel B of Fig. 1, in which the analysis is limited to workers classified as mismatched. It can be shown that 19% of those classified as mismatched have a distance measure between 0.6 and 0.7, but all points of the distribution are well represented (except the one corresponding to zero). About 15% of mismatched individuals have occupations with quite similar activities to the ones in their corresponding fields of study, with distance measures between 0.0 and 0.2. However, the interval between 0.8 and 1, which indicates a high degree of dissimilarity between area of study and occupation, also contains 15% of mismatched individuals.^11^

Panel C of Fig. 1 reports the distribution of matched workers. It can be seen that most of the individuals in this group have occupations quite similar to their areas of study, and that the distance measure is zero for three quarters of them. A distance measure greater than  0.2 is an unusual situation among matched workers.

**Fig. 2:**
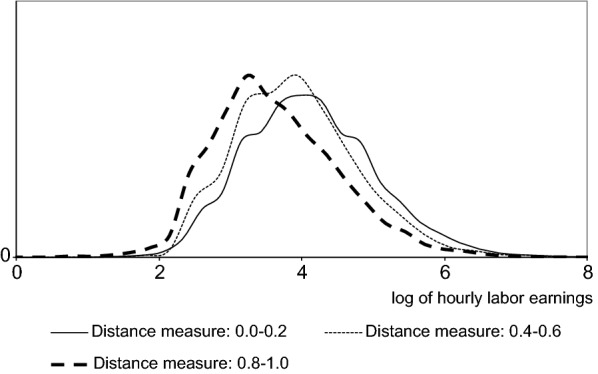
Kernel density estimates of hourly labor earnings among mismatched workers by intervals of the distance measure. The sample comprises employed individuals with a bachelor’s degree, aged between 25 and 60 years, classified as mismatched. Source: the 2010 Brazilian census

The heterogeneity of distance measures helps to explain part of the large dispersion in hourly labor earnings among workers classified as mismatched.^12^ Figure 2 reports the density of hourly labor earnings distribution for mismatched individuals arranged into three groups according to the distance measure between their fields of study and occupations. Labor earnings distributions indicate a much better situation for those with distance measure between 0 and 0.2 than for those for whom the distance measure ranges between 0.4 and 0.6. However, the latter group has a better situation compared to that of individuals with occupations that are quite different from their areas of study.

## Labor earnings and distance measure between workers’ fields of study and occupations

### Empirical approach

Following Robst (2007)’s seminal paper, the labor earnings penalty associated with mismatches between occupation and field of study has been usually estimated by the following Mincer-type earnings equation:

3 $$\ln \left( {w_{i} } \right) = \beta_{0} + \beta_{1} X_{i} + \beta_{2} F_{i} + \beta_{3} M_{i} + \beta_{4} P_{i} + u_{i} .$$

where *w*_*i*_ represents hourly labor earnings, *X*_*i*_ is a vector of demographic characteristics, *F*_*i*_ represents dummy variables for fields of study, *M*_*i*_ is a dummy equal to 1 for individuals in occupations unrelated to their fields of study, and equal to 0 otherwise, *P*_*i*_ denotes a dummy for those partially matched, and *u*_*i*_ is the error term. The dummies for fields of study refer to 10 aggregated groups, whereas demographic characteristics are represented by age, age squared, gender, race, and dummies for region of residence. Penalties associated with being mismatched or partially matched are represented by coefficients *β*_3_ and *β*_4_.

In order to investigate whether differences between skill contents required by the occupation and those acquired in tertiary education provide additional information on the way the job-education mismatch is related to labor earnings, the distance measure (*Dist*_*i*_), as stated in Eq. (2), is included as independent variable in the labor earnings equation.

4 $$\ln \left( {w_{i} } \right) = \beta_{0} + \beta_{1} X_{i} + \beta_{2} F_{i} + \beta_{3} M_{i} + \beta_{4} P_{i} + \beta_{5} Dist_{i} + e_{i} .$$

Equation (4) is also estimated by excluding *M*_*i*_ and *P*_*i*_, that is, by using only *Dist*_*i*_ to represent the mismatch between area of study and occupation. As a robustness check, regressions are also estimated using Dist*, whose construction is explained in Sect. 2.3, instead of *Dist* as the independent variable. All regressions are estimated by OLS for the total sample and separately for men and women, since empirical evidence on the consequences of the field of education-occupation mismatch usually shows different results by gender.^13^

It is important to mention that estimates of Eqs. (3) and (4) may be biased because of the possible correlation between mismatch variables and omitted factors. Mismatched workers may be less able than those adequately matched, and the labor earnings penalty may reflect this ability differential. Unfortunately, the 2010 census does not provide any variable that can proxy for ability. Evidence provided by Lemieux (2014) and Nordin et al. (2010), however, shows that the inclusion of proxy variables for ability has no impact on the estimated effect of mismatch variables on labor earnings.

### Evidence on the relationship between mismatch and hourly labor earnings

Column (1) of Table 2 shows that workers classified as mismatched earn 27% (exp(− 0.318) − 1 = − 0.272) less than those in occupations related to their fields of study, controlling for demographic characteristics and fields of study. Also according to estimates, hourly labor earnings of weakly matched workers are not statistically lower than those received by matched individuals.

**Table 2:** Hourly labor earnings and the occupation-field of study mismatch

	Sample				
	Total			Mismatched	Matched
	(1)	(2)	(3)	(4)	(5)
Mismatch	− 0.318 [8.81]***	− 0.076 [1.84]*			
Weak match	− 0.061 [1.25]	− 0.002 [0.03]			
Distance		− 0.505 [11.23]***	− 0.592 [13.23]***	− 0.61 [19.07]***	− 0.124 [0.67]
Age	0.065 [19.22]***	0.063 [18.72]***	0.062 [18.52]***	0.063 [28.92]***	0.071 [9.84]***
Age squared	− 0.001 [14.69]***	− 0.001 [14.17]***	− 0.001 [13.89]***	− 0.001 [20.56]***	− 0.001 [7.73]***
Female	− 0.25 [16.52]***	− 0.256 [16.93]***	− 0.257 [16.37]***	− 0.254 [23.61]***	− 0.232 [6.60]***
Black	− 0.214 [24.10]***	− 0.205 [24.66]***	− 0.205 [24.84]***	− 0.211 [35.76]***	− 0.199 [11.75]***
Constant	2.492 [39.13]***	2.539 [39.33]***	2.532 [44.07]***	2.58 [45.69]***	2.351 [15.70]***
Observations	554,439	554,439	554,439	313,128	171,647

The dependent variable is the logarithm of the hourly labor earnings

All regressions include regional dummies and dummies for fields of study

Regressions are estimated by OLS and robust t-statistics are in brackets

Standard errors are clustered at the occupation-field of study level

*, **, ***—indicate significance on the 10%, 5% and 1% level respectively

The estimated earnings penalty for mismatched workers reported in Table 2 is larger than that reported by college/university graduates in the USA (Robst 2007) and in Sweden (Nordin et al. 2010). According to Robst (2007), annual earnings of mismatched individuals are 11% lower compared to those having a major subject that matches their occupation, while Nordin et al. (2010)’s estimates indicate that the earnings penalty is about 20%. Both papers also provide evidence that weakly matched workers usually earn 2% less than matched ones. Fernandes and Narita (2001), using data from the 1980 and 1991 Brazilian censuses, show that individuals in occupations closely related to their fields of study earn 13% more than mismatched ones.^14^

In column (2), the distance measure and the dummies for mismatch and weak match are included at the same time as independent variables in the hourly labor earnings equation. In this case, the estimated coefficient for the mismatch dummy drops (in absolute value) from − 0.318 to − 0.076, while the coefficient for weak match drops from − 0.061 to − 0.003 and remains non-significant. So, adding the distance measure to the set of independent variables, the coefficient for mismatch dummy is about 75% smaller relative to the result reported in column (1). Also, the distance measure seems to be related to important changes in hourly labor earnings. Evidence indicates that a 0.10 increase in the distance measure, which ranges between 0 and 1, is associated with 5% lower labor earnings.

Regression in column (3) includes the distance measure between field of study and occupation, but it excludes the two dummy variables indicating the mismatch status. According to the estimated coefficient associated with the distance measure, labor earnings diminish 6% for each 0.10 increase in *Dist*_*i*_. In column (4), the sample is restricted to mismatched individuals, and the estimated coefficient for the distance measure is quite similar to the one in column (3). Restricting the analysis to matched workers, the estimated coefficient for the distance measure becomes non-significant [column (5)].

Appendix Table 8 shows regressions analogous to those reported in Table 2 but representing the distance between field of study and occupation by the alternative measure described in Sect. 2.3 (Dist*). The estimated coefficients associated with Dist* are larger in absolute value compared to the ones in columns (2) and (3) of Table 2. Also, the estimated drop of the mismatch dummy coefficient between columns (1) and (2) is even more pronounced in Appendix Table 8 than in Table 2.

Evidence indicates the distance measure helps to explain differences in labor earnings that the dummies for mismatched and weakly matched individuals are not able to do. According to column (1) of Table 2, a computer scientist who works as a telephone switchboard operator, for example, has an earnings penalty equal to 27% relative to an individual with the same degree who works as a systems analyst, which is classified as a close match between field of study and occupation, controlling for the characteristics included in the regression. The estimated earnings penalty is the same for another computer scientist who works as a physical and engineering science technician, for example. The comparison using estimates from column (1) does not take into account how activities performed by mismatched workers in their occupations are related to activities associated with their corresponding areas of study. The distance measure for a computer scientist who works as a telephone switchboard operator is 0.560, but 0.202 for an individual with the same degree who works as a physical and engineering science technician. Taking into account the estimated coefficient for *Dist*_*i*_ in column (3), the earnings penalty for the former individual is 30%. For the latter individual, however, the estimated earnings penalty is about 10%. In both cases, the estimated penalties associated with *Dist*_*i*_ represent one-third of the actual difference between mean hourly labor earnings for computer scientists in each of those two occupations relative to an appropriately matched computer scientist.

Repeating the same exercise described above with values from Appendix Table 7 for the closest and most distant matches provides remarkable differences. The estimated labor earnings penalty is almost 50% for an individual with a degree in accounting who works as a mixed crops grower, whereas there is no earnings penalty for another individual with a degree in economics who works as a supply, distribution and related manager, although both can be classified as mismatched.

Table 3 presents the estimated results for Eq. (1) separately by gender. In columns (1) and (4), being mismatched is associated with an earnings penalty slightly more pronounced for women than for men. In addition, being weakly matched relative to having an occupation related to the field of study is associated with a drop in hourly labor earnings only for women (12%). Both Robst (2007) and Nordin et al. (2010) also show that women are more penalized as a consequence of field of study mismatch than men, mainly in the latter study.

**Table 3:** Hourly labor earnings and the occupation-field of study mismatch by gender

	Male			Female		
	(1)	(2)	(3)	(4)	(5)	(6)
Mismatch	− 0.305 [7.91]***	− 0.048 [0.98]		− 0.337 [9.11]***	− 0.103 [2.54]**	
Weak match	0.032 [0.62]	0.119 [2.12]**		− 0.12 [2.64]***	− 0.075 [1.57]	
Distance		− 0.54 [10.34]***	− 0.606 [12.50]***		− 0.485 [10.36]***	− 0.588 [12.91]***
Age	0.08 [30.00]***	0.078 [30.33]***	0.078 [30.30]***	0.053 [13.20]***	0.051 [12.81]***	0.05 [12.38]***
Age squared	− 0.001 [25.20]***	− 0.001 [25.38]***	− 0.001 [25.01]***	− 0.001 [9.23]***	− 0.001 [8.80]***	− 0.001 [8.37]***
Female						
Black	− 0.226 [33.62]***	− 0.215 [34.37]***	− 0.215 [34.04]***	− 0.202 [15.25]***	− 0.195 [15.32]***	− 0.195 [15.92]***
Constant	2.152 [35.41]***	2.21 [35.66]***	2.231 [38.36]***	2.535 [33.38]***	2.577 [33.98]***	2.536 [36.28]***
Observations	246,588	246,588	246,588	307,851	307,851	307,851

The dependent variable is the logarithm of the hourly labor earnings. All regressions include regional dummies and dummies for fields of study. Regressions are estimated by OLS and robust

t-Statistics are in brackets

Standard errors are clustered at the occupation-field of study level

*, **, ***—indicate significance on the 10%, 5% and 1% level respectively

As shown in column (2), the coefficient associated with the weak match indicator becomes positive and significant in the specification that includes the distance measure as independent variable for men, whereas the earnings penalty for a mismatch becomes non-significant.^15^ Estimates for women that add the distance measure in column (5) show that the coefficients for mismatch and weak match dummies drop to around one-third and two-thirds of the values reported in column (4), and only the coefficient for mismatch remains statistically significant.

Columns (3) and (6) of Table 3 report the results of regressions that include the distance measure instead of binary variables to represent the mismatch between field of study and occupation. The estimated coefficients associated with the distance measure are almost the same for both men and women and similar to those reported in column (3) of Table 2, using all individuals in the sample, which indicates that a 0.1 increase in the distance measure is related to a 6% drop in hourly labor earnings.

Table 4 presents the estimates of Eqs. (3) and (4) separately by field of study. Column (1) shows the estimated coefficients for the mismatch dummy in a specification based on versions of Eq. (3) that exclude *F*_*i*_. Labor earnings penalties for working in an occupation unrelated to the area of study are more pronounced for those who completed a degree in health professions or sciences, mathematics, and computing programs, while the estimated effect is non-significant in humanities and arts.

**Table 4:** Hourly labor earnings and mismatch by field of study

	Equation (3)	Equation (4)	
	Estimated coefficient for the mismatch dummy	Estimated coefficient for the mismatch dummy	Estimated coefficient for the distance measure
	(1)	(2)	(3)
Education	− 0.258 (6.25)***	0.028 (0.42)	− 0.590 (6.53)***
Humanities and arts	− 0.118 (1.15)	0.125 (1.13)	− 0.437 (7.07)***
Social sciences	− 0.211 (4.80)***	0.075 (1.08)	− 0.619 (6.47)***
Business	− 0.309 (5.96)***	− 0.049 (0.80)	− 0.572 (8.52)***
Law	− 0.272 (5.79)***	0.335 (1.70)*	− 0.921 (3.41)***
Science, mathematics and computing	− 0.430 (8.26)***	− 0.329 (4.02)***	− 0.367 (4.05)***
Engineering	− 0.266 (5.58)***	− 0.022 (0.42)	− 0.577 (11.52)***
Agriculture and veterinary	− 0.151 (3.62)***	0.087 (1.08)	− 0.551 (5.43)***
Health	− 0.520 (3.47)***	− 0.201 (1.96)**	− 0.623 (5.57)***
Services	− 0.249 (8.60)***	− 0.028 (0.63)	− 0.510 (6.49)***

The dependent variable is the logarithm of the hourly labor earnings. All regressions include age, age squared, a dummy for female, a dummy for black and regional dummies. Regressions are estimated by OLS and t-statistics are in brackets

Standard errors are clustered at the occupation-field of study level

*, **, ***—indicate significance on the 10%, 5% and 1% level respectively

Differences across areas of study in column (1) of Table 4 are quite similar to the ones reported by Robst (2007) for the US. According to Robst (2007), those differences are a consequence of the fact that the level of mismatch is greater in programs that teach occupation-specific skills.

Adding the distance measure to the regressions, the coefficients for the mismatch dummy become non-significant for most of the areas in column (2) of Table 4. The estimated coefficients associated with the distance measure in column (3) are negative and significant at the 1% level for all fields of study, ranging between − 0.37 (sciences, mathematics, and computing) and − 0.92 (law).

**Fig. 3:**
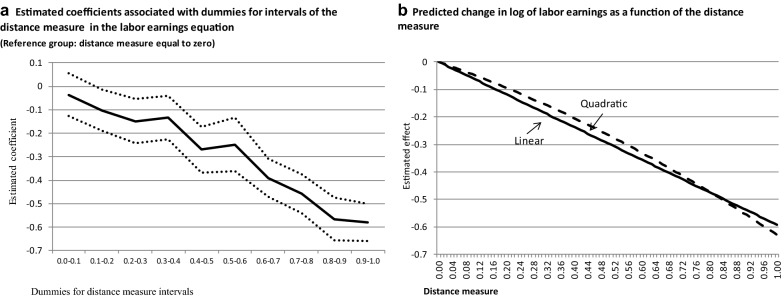
Hourly labor earnings and the occupation-field of study mismatch: non-linear specifications. Note: The dependent variable is the logarithm of the hourly labor earnings. All regressions are estimated by OLS and control for age, age squared, a dummy for female, a dummy for black, regional dummies and dummies for fields of study

In order to investigate whether the relationship between log of labor earnings and the distance measure is non-linear, Fig. 3a plots the estimated coefficients associated with dummies for 10 intervals of the distance measure in a regression similar to those in columns (1)–(3) of Table 2, but representing mismatch by that set of dummies. The reference group is represented by those for whom the measure is equal to 0. According to the results, there is no income penalty for workers for whom the distance measure is higher than 0 but lower than 0.1, whereas those with a distance measure between 0.1 and 0.2 have a labor earnings penalty equal to 10%, which is equivalent to the earnings reduction associated with a distance measure equal to 0.17, according to column (3) of Table 2. Among those in occupations that are quite different from their fields of study, for whom the measure falls on the interval between 0.9 and 1.0, the estimated coefficient in Fig. 3a indicates a 44% reduction in labor earnings. This value corresponds to an earnings penalty associated with a distance measure of 0.98 in column (3) of Table 2.

Figure 3b shows the predicted change in log of labor earnings as a function of the distance measure, using the estimated coefficient in column (3) of Table 2, and the estimated coefficients for distance and distance squared in a specification where this latter variable is included in the model.^16^ Both Fig. 3a, b indicate that the relationship between the distance measure and hourly labor earnings seems to be linear.

## Conclusions

Students at university/college usually learn skills necessary to work in an occupation or in a small set of occupations directly related to the chosen field of study. Although those individuals acquire general skills, most of the skills learned at this educational level are occupation-specific. For those graduate workers who have occupations unrelated to their areas of study, it is possible that a greater portion of the skills acquired during tertiary education is not useful in the labor market.

The task approach to labor market distinguishes between task, a unit of work activity that produces output, and skill, a worker’s endowment of capabilities for performing various tasks. Workers’ skills are applied to tasks to generate output. This framework seems very appropriate for investigating the mismatch between field of study, which is related to the skills acquired during tertiary education, and occupation, which can be represented by a number of activities performed by workers. Mismatch can be characterized as a situation where workers’ skills do not correspond to skills required to perform tasks in their occupations.

Even among mismatched workers with the same degree, skills acquired at university/college do not transfer in the same way to all occupations. The job task approach allows a better characterization of the degree of transferability of skills through the relationship between activities performed in an occupation directly related to the worker’s field of study and those made in his or her actual occupation than the simple categorization of workers as matched, weakly matched, and mismatched.

This paper intends to construct a continuous measure to represent the distance between skills acquired in tertiary education and those required in an individual’s occupation. This measure is based on the similarity between activities usually performed in each individual’s occupation and those required in the occupation considered the most closely related to his or her field of study. The distance measure is computed empirically by combining data from the 2010 Brazilian census and descriptions of occupations from the 2010 CBO.

According to the evidence presented in this paper, workers classified as mismatched could be quite heterogeneous. Among mismatched workers, there are individuals with distance measure close to zero, as well as those in occupations with activities completely different from activities performed in occupations related to their fields of study, for whom the distance measure is equal to one.

Estimates also show the degree of relatedness between area of study and occupation seems to be important to understand labor earnings differences between matched and mismatched workers, as well as among those classified as mismatched. Despite labor earnings penalties associated with being mismatched in most of the cases, individuals who have occupations with similar skills to those acquired in their fields of study earn more than workers who have occupations largely unrelated to their fields of study. Therefore, the results presented here emphasize the importance to consider more accurate descriptions of workers and occupations to better characterize the occupation-field of study mismatch and its consequences for labor earnings.

## Appendix

See Tables 5, 6, 7, and 8.

**Table 5:** Summary statistics of tasks across 4-digit occupations in the 2010 CBO

Task groups	Mean	Standard deviation
1—Research, analyze, evaluate	0.060	0.118
2—Plan, construct, design, sketch	0.110	0.134
3—Execute laws or interpret rules	0.014	0.075
4—Negotiate, coordinate, organize, employ and manage personnel	0.206	0.225
5—Teach or train others, consult, inform	0.029	0.065
6—Sell, buy, advise customers, advertise	0.055	0.103
7—Entertain, present, and publish	0.012	0.068
8—Calculate, bookkeeping	0.016	0.058
9—Correct texts or data	0.034	0.079
10—Measure, quality control	0.076	0.104
11—Operate or control machines	0.050	0.116
12—Repair or renovate machines/vehicles	0.042	0.093
13—Cultivate	0.039	0.139
14—Manufacture, extract, mold materials, build	0.154	0.233
15—Cleaning	0.021	0.057
16—Pack, ship, transport	0.042	0.097
17—Serve or accommodate, treat others	0.029	0.090
18—Secure	0.011	0.054

Calculations based on the 2010 CBO

**Table 6:** The field of study-occupation matches

Field of study	Occupations that match the field of study their field of education
Teacher training and education science (general programmes)	*Education methods specialists*
Education science	*Education methods specialists*, education managers
Training for pre-school teachers	*Early childhood educators*
Training for teachers at basic levels	*Primary school teachers*, special needs teachers
Training for teachers with subject specialisation	*Secondary education teachers*
Training for teachers of vocational subjects	*Vocational education teachers*, arts teachers
Arts (general programmes)	*Visual artists, graphic and multimedia designers and creative and performing artists*, arts teachers
Fine arts	*Visual artists, graphic and multimedia designers and creative and performing artists*, arts teachers
Music and performing arts	Other arts teachers; *musicians, singers and composers*; film, stage and related directors and producers; actors
Audio-visual techniques and media production	*Visual artists, graphic and multimedia designers and creative and performing artists*
Design	*Designers*
Craft skills	*Visual artists, graphic and multimedia designers and creative and performing artists*
Religion	*Religious professionals*
Foreign languages	*Language teachers*; translators, interpreters and other linguists
Mother tongue	*Language teachers*
History and archaeology	Archivists and museologists; *philosophers, historians and political scientists*
Philosophy and ethics	*philosophers, historians and political scientists*
Social and behavioural science (general programmes)	*Sociologists, anthropologists and related professionals*
Psychology	*Psychologists*
Sociology and cultural studies	*Sociologists, anthropologists and related professionals*
Political science and civics	*Philosophers, historians and political scientists*
Economics	Financial analysts; *economists*
Journalism and reporting	Authors and related writers; *journalists*; announcers on radio, television and other media
Library, information, archive	*Librarians and related information professionals*; archivists and museologists
Business and administration (general programmes)	Business services and administration managers; sales and marketing managers, advertising and marketing professionals, public relations professionals and sales professionals; *administration professionals*
Wholesale and retail sales	*Retail and wholesale trade managers*; sales and marketing managers
Marketing and advertising	Advertising and public relations managers; *advertising and marketing professionals, public relations professionals and sales professionals*; sales and marketing managers
Finance, banking, insurance	Finance managers; financial and insurance services branch managers; *financial analysts*
Accounting and taxation	*Accountants*
Management and administration	*Managing directors and chief executives*; agricultural and forestry production managers; manufacturing managers and mining managers; construction managers; supply, distribution and related managers; child care services managers, aged care services managers, social welfare managers and sports, recreation and cultural centre managers; health services managers; education managers; hotel and restaurant managers; services managers; human resource professionals; administration professionals; human resource managers; retail and wholesale trade managers; financial and insurance services branch managers
Working life	*Human resource professionals*
Law	*Lawyers*; judges; legal professionals
Life science (general programmes)	*Biologists, botanists, zoologists and related professionals*
Biology and biochemistry	*Biologists, botanists, zoologists and related professionals*
Environmental science	*Biologists, botanists, zoologists and related professionals*
Physical science	*Physicists and astronomers*; meteorologists; geologists and geophysicists
Physics	*Physicists and astronomers*
Chemistry	*Chemists*
Earth science	Physicists and astronomers; meteorologists; *geologists and geophysicists*
Mathematics	*Mathematicians*
Statistics	*Statisticians*
Computer science	Database and network professionals; *systems analysts*
Computer use	Database and network professionals; *systems analysts*
Data processing	*Database and network professionals*; systems analysts
Engineering and engineering trades (general programmes)	Electrical and electronics engineers; *mechanical engineers*; mining engineers, metallurgists and related professionals; cartographers and surveyors
Mechanics and metal work	*Mechanical engineers*; mining engineers, metallurgists and related professionals
Electricity and energy	*Electrical and electronics engineers*
Electronics and automation	*Electrical and electronics engineers*
Chemical and process	*Chemical engineers*
Motor vehicles, ships and aircraft	*Mechanical engineers*
Manufacturing and processing (general programmes)	*Industrial and production engineers*
Food processing	*Industrial and production engineers*
Textiles, clothes, footwear, leather	*Industrial and production engineers*
Materials (wood, paper, plastic, glass)	*Industrial and production engineers*
Mining and extraction	*Mining engineers, metallurgists and related professionals*
Architecture and town planning	*Building architects and town planners*
Building and civil engineering	*Civil engineers*
Agriculture, forestry and fishery (general programmes)	*Farming, forestry and fisheries advisers*
Crop and livestock production	*Farming, forestry and fisheries advisers*
Horticulture	*Farming, forestry and fisheries advisers*
Forestry	*Farming, forestry and fisheries advisers*
Fisheries	*Farming, forestry and fisheries advisers*
Veterinary	*Veterinarians*
Health (general programmes)	Physiotherapists; *nursing professionals*; dieticians and nutritionists; audiologists and speech therapists
Medicine	*Medical doctors*
Nursing and caring	*Nursing professionals*; environmental and occupational health and hygiene professionals
Dental studies	*Dentists*
Medical diagnostic and treatment technology	*Nursing professionals*
Therapy and rehabilitation	*Physiotherapists*; nursing professionals; dieticians and nutritionists; audiologists and speech therapists
Pharmacy	*Pharmacists*
Child care and youth services	*Social work and counselling professionals*
Social work and counselling	*Social work and counselling professionals*
Personal services (general programmes)	*Child care services, aged care services, social welfare and sports, recreation and cultural centre managers*
Hotel, restaurant and catering	*Hotel and restaurant managers*
Travel, tourism and leisure	*Hotel and restaurant managers*
Sports	*Child care services, aged care services, social welfare and sports, recreation and cultural centre managers*
Domestic services	*Child care services, aged care services, social welfare and sports, recreation and cultural centre managers*
Hair and beauty services	*Child care services, aged care services, social welfare and sports, recreation and cultural centre managers*
Transport services	*Supply, distribution and related manager*

Occupations used to represent tasks in each field of study are in italics

**Table 7:** Measuring distances between fields of study and occupations among mismatched workers. Source: the 2010 census

Field of study	Occupation	Distance measure
Most similar comparisons		
Education science	Health and child care services managers	0.004
Economics	Supply, distribution and related managers	0.005
Business and administration	Manufacturing managers	0.006
Computer science	Administrative and executive secretaries	0.009
Business and administration	Financial and insurance services branch managers	0.010
Most distant comparisons		
Management	Mixed crop growers	1.000
Accounting	Mixed crop growers	1.000
Training for teachers with subject specialisation	Manufacturing labourers not elsewhere classified	1.000
Management	Metal processing plant operators	1.000
Law	Stock clerks	1.000
Most common associations between field of study and occupation among mismatched		
Management	General office clerks	0.674
Mother tongue	Primary school teachers	0.708
Management	Shop sales assistants	0.659
Management	Administrative and executive secretaries	0.234
Law	Legal and related associate professionals	0.660

Only matches with at least 50 observations are considered

**Table 8:** Hourly labor earnings and the occupation-field of study mismatch

	Sample				
	Total			Mismatched	Matched
	(1)	(2)	(3)	(4)	
Mismatch	− 0.318 [8.81]***	− 0.056 [1.62]			
Weak match	− 0.061 [1.25]	− 0.002 [0.03]			
Distance*		− 0.577 [16.52]***	− 0.642 [14.40]***	− 0.61 [20.03]***	− 0.713 [1.63]
Age	0.065 [19.22]***	0.062 [18.73]***	0.062 [18.49]***	0.063 [29.37]***	0.07 [10.01]***
Age squared	− 0.001 [14.70]***	− 0.001 [14.14]***	− 0.001 [13.81]***	− 0.001 [20.78]***	− 0.001 [7.81]***
Female	− 0.25 [16.52]***	− 0.252 [16.89]***	− 0.252 [16.55]***	− 0.249 [22.50]***	− 0.229 [7.17]***
Black	− 0.214 [24.10]***	− 0.202 [25.54]***	− 0.202 [26.03]***	− 0.208 [36.04]***	− 0.196 [13.10]***
Constant	2.492 [39.13]***	2.56 [40.74]***	2.556 [46.14]***	2.578 [47.82]***	2.391 [16. 3 0]***
Observations	554,488	554,488	554,488	313,177	171,647

The dependent variable is the logarithm of the hourly labor earnings

All regressions include regional dummies and dummies for fields of study

Regressions are estimated by OLS and robust t-statistics are in brackets

Standard errors are clustered at the occupation-field of study level

*, **, ***—indicate significance on the 10%, 5% and 1% level respectively

## Footnotes

1 Robst (2007), (Nordin et al. 2010) and Lemieux (2014) provide evidence for developed countries that workers tend to get a wage penalty when they have a job in an occupation that is not matched to their fields of study. Estimates from Brazil show similar results to those reported from developed countries (Fernandes and Narita 2001; Reis and Machado 2016).

2 See Acemoglu and Autor (2011) and Autor (2013) for surveys on task approach to labor markets.

3 These 18 task categories are similar to the 19 categories defined in the German Qualification and Career Survey (see Gathmann and Schönberg 2010; Spitz-Öener 2006).

4 According to Eq. (1), the weights of tasks performed by an electronic engineer are the following: researching and analyzing (0.14), planning and designing (0.72), coordinating and organizing (0.14), and all other tasks have a weight equal to 0, while the results for a sales worker are: coordinating and organizing (0.11), selling (0.45), calculating (0.11), performing quality control (0.11), packing and transporting (0.11), serving and accommodating (0.11), and weights are equal to 0 for other tasks.

5 Robst (2007) uses self-reported information on whether workers classify their occupations as closely related, somewhat related, or unrelated to their fields of study. Lemieux (2014) makes use of a similar question to construct average measures at the occupation-field of study level. As mentioned by Robst (2007), responses to those questions have a large degree of subjectivity.

6 The empirical approach assumes that an individual who completed a degree in mechanical engineering accumulated human capital to perform those activities related to mechanical engineers according to the 2010 CBO. If this same individual works as a civil engineer, it is assumed that he or she is ready to carry out activities that are also performed by mechanical engineers, but not the other activities associated with his or her occupation.

7 Gathmann and Schönberg (2010) use this measure to characterize the skill requirement distance between two occupations.

8 The aim of the distance measure is to represent general features of the transferability of skills, although there are aspects that are specific to occupations and areas of study.

9 The larger proportions of women and blacks contribute to lower average labor earnings among weakly matched workers.

10 By definition, the distance measure is equal to 0 when the individual’s occupation is also the one defined as the most closely related to his/her field of study. In the same way, an individual can be classified as matched, although his/her distance measure is higher than 0.

11 Appendix Table 7 shows the closest and the most distant field of study-occupation pairs among workers classified as mismatched. Appendix Table 7 also presents the most common field of study-occupation pairs and their corresponding distance measures.

12 The Theil index is 0.85 for mismatched workers and equal to 0.58 and 0.59 for matched and weakly matched ones.

13 Also, the distribution of women across fields of study, as well as across occupations, is quite different from that of men.

14 The sample in Fernandes and Narita (2001) includes only those in urban areas who worked 15 or more hours in the week of reference of the survey. The period of reference for occupation and labor earnings in the 1980 and 1991 censuses is also different from that in the 2000 and 2010 censuses.

15 Weak match for male workers is associated with promotion to occupations that usually pay higher earnings, such as a civil engineer who works as a construction manager, for example. Note that a distance measure equal to 0.2 is enough to cancel the gain related to being weakly matched in column (2).

16 The coefficient for the distance squared is non-significant. A model representing the distance by a cubic polynomial produces results almost identical to the ones provided by the model with distance squared.
